# Evaluation of Physicochemical Characteristics in Drinking Water Sources Emphasized on Fluoride: A Case Study of Yancheng, China

**DOI:** 10.3390/ijerph16061030

**Published:** 2019-03-21

**Authors:** Yumin Wang, Ran Yu, Guangcan Zhu

**Affiliations:** School of Energy and Environment, Southeast University, Nanjing 210096, China; wangyumin@seu.edu.cn (Y.W.); yuran@seu.edu.cn (R.Y.)

**Keywords:** fluoride content, health risk assessment, correlation analysis, cluster analysis, factor analysis, physicochemical characteristics

## Abstract

In this study, the concentration of fluoride and the associated health risks for infants, children, and adults were analyzed and compared for three drinking water sources in Yancheng City, Jiangsu Province, China. To analyze the relationship between the water quality parameters of pH, fluoride (F^−^), sulfate (SO_4_^2−^), chloride (Cl^−^), total dissolved solids (TDS), total alkalinity (TAlk), sodium (Na^+^), and potassium (K^+^), statistical analyses including correlation analysis, R-mode cluster analysis and factor analysis were performed based on monthly data from the year 2010 to 2015. The results indicated: (1) Fluoride concentrations in the drinking water sources ranged from 0.38 to 1.00 mg L^−1^ (mean = 0.57 mg L^−1^) following the order of Tongyu River > Yanlong Lake > Mangshe River; (2) fluoride concentrations in 22.93% of the collected samples were lower than 0.5 mg L^−1^, which has the risk of tooth cavities, especially for the Mangshe River; (3) the fluoride exposure levels of infants were higher than children and adults, and 3.2% of the fluoride exposure levels of infants were higher than the recommended toxicity reference value of 122 μg kg^−1^ d^−1^ as referenced by Health Canada, which might cause dental fluorosis issues; (4) the physico-chemical characteristics are classified the into four groups reflecting F^−^- TAlk, Na^+^-K^+^, SO_4_^2−^-Cl^−^, and pH-TDS, respectively, indicating that fluoride solubility in drinking water is TAlk dependent, which is also verified by R-mode cluster analysis and factor analysis. The results obtained supply useful information for the health department in Yancheng City, encouraging them to pay more attention to fluoride concentration and TAlk in drinking water sources.

## 1. Introduction

Fluoride (F^−^) enters the water body as a result of the geological composition of bedrock and soil, as well as dental products, food, combustion of fluoride-rich coal, pharmaceuticals, and pesticides. It is generally accepted that human beings consume fluoride largely from drinking water, and about 90% of fluoride that exists in drinking water is absorbed by the digestive system [1,2,3,4]. Fluoride deficiency or excess has an adverse effect on human health. Much work had been carried out on the effects of high fluoride concentration in drinking water, which may cause skeletal issues on both bones and teeth, especially for children and pregnant women [3,4,5,6,7,8]. Since fluoride concentration beyond 4 mg L^−1^ may induce skeletal fluorosis, and beyond 10 mg L^−1^ may cause crippling skeletal fluorosis, the U.S. Environmental Protection Agency (EPA) has set the maximum contaminant level (MCL) for fluoride in drinking water as 4 mg L^−1^, and the secondary maximum contaminant level (SMCL) as 2 mg L^−1^. According to Chinese sanitary standards, the fluoride content for drinking water sources is 1.0 mg L^−1^ (MHPRC, 2006). Although the lower limit of fluoride content in water sources is not specified, fluoride content of lower than 0.5 mg L^−1^ will increase the risk of dental caries, affect the formation of dental enamel, as well as decrease the mineralization of bones, especially for children who are in the body growth stage [9,10,11,12]. Since the 1940s, fluoridation schemes were introduced into the United States, England, and Wales with the objective of improving dental health, which decreased the proportion of decayed, missing and filled teeth in children significantly. Additionally, fluoridated toothpaste and fluoride-containing mouthwash can also contribute to fluoride intake in children [2,13]. The World Health Organization recommends F^−^ concentrations in drinking water which range from 0.5 mg L^−1^ to 1.5 mg L^−1^ for tooth deterioration prevention. The US Public Health Service (USPHS) recently recommend optimal fluoride concentrations of 0.7 mg L^−1^ in community water systems, which can prevent dental caries but also lower the risk of dental fluorosis [12,14]. Since low and high contents of fluoride in drinking water both pose threats on human health, it is of great importance to evaluate the F^−^ content in drinking water sources accurately [15,16]. 

This study aimed to analyze and compare fluoride content and the associated health risks for infants, children, and adults in three drinking water sources of Yancheng City: the Tongyu River, Yanlong Lake, and Mangshe River. Moreover, the relationship between the other physicochemical parameters of pH, fluoride (F^−^), sulfate (SO_4_^2−^), chloride (Cl^−^), total dissolved solids (TDS), total alkalinity (TAlk), sodium (Na^+^), and potassium (K^+^) were investigated, summarized, and analyzed by multivariate statistical analysis method.

## 2. Materials and Methods 

### 2.1. Study Area

**Figure 1:**
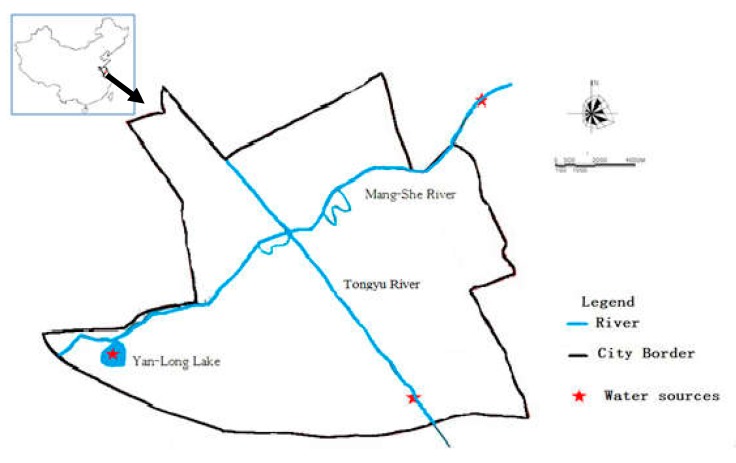
The drinking water sources in Yancheng City.

Yancheng (119°34′–120°27′ E, 32°51′–34°12′ N) is an eastern coastal city in the center of the Jiangsu province of China with a population of over 8 million. It faces the Yellow Sea on the east, and is to the west of Yangzhou City and Huai’an City, the north of Lianyungang City, and the south of Nantong and Taizhou cities. The research area is 48.54 km^2^, shown in Figure 1, with three drinking water sources: the Tongyu River, Mangshe River, and Yanlong Lake. The Tongyu River has a total length of 415 km, originating from the Changjiang River, and ends its course in Lianyungang City. The middle reach of the Tongyu River runs through Yancheng City with a length of 183.6 km, and has an average flow of approximately 100 m^3^/s. The Mangshe River originates from Dazong Lake and discharges into the East Sea with a total length of nearly 50 km. The Tongyu River and the Mangshe River serve the complex functions of drinking water sources, fairways, irrigation water sources, and flood drainage system. The Yanlong Lake is a man-made ecological lake built in 2012 occupying 2.23 km^2^ with pretreatment area, ecological wetland purification area, and deep purification area, which also serves as a drinking water source for Yancheng City [17]. Up to now, several water pollution events have occurred in the drinking water sources of Yancheng City. In 2009, sewage from chemical plants with volatile phenolic compounds were illegally drained upstream of water sources, resulting in the cutting off of the water supply for as long as 66 hours, which severely affected the life of nearly 200,000 inhabitants as well as industrial processes. Therefore, the water sources of Yancheng city should be monitored closely and paid more attention to, especially as regards the water quality parameters related to human health.

### 2.2. Samples and Measurements

One hundred and fifty-six samples, including 60 samples in the Tongyu River from January, 2010 to December, 2015, 54 samples in the Mangshe River from July, 2011 to Decemeber, 2015, and 42 samples in Yanlong Lake from July, 2012 to December, 2015, were collected during the study. The GPS coordination of sample sites are (120°02′6″ E, 33°32′1″ N), (120°04′39″ E, 33°21′56″ N), and (120°01′36″ E, 33°19′59″ N) for the Tongyu River, Mangshe River, and Yanlong Lake, respectively. Parameters of pH, fluoride (F^−^), sulfate (SO_4_^2−^), chloride (Cl^−^), total dissolved solids (TDS), total alkalinity (TAlk), sodium (Na^+^) and potassium (K^+^) were analyzed using the standard water analysis methods (seen in Table 1). The selected parameters of pH, SO_4_^2−^, Cl^−^, TDS, Na^+^, and K^+^ were reported to be related to fluoride concentration [5,18,19], and parameters of Ca^2+^ and Mg^2+^ had a minor relationship with fluoride concentration [19]. Moreover, TDS represents the sum of cations and anions as the principal inorganic constituents. The parameters were measured in accordance with the standard methods. 

### 2.3. Health Risk Assessment

In order to measure the variation of the health risks caused by exposure to fluoride, the health risk assessment model was applied to simulate the health effects of fluoride as follows [20]. The population was divided into three age groups based on physiological and behavioral differences: infants, children, and adults.

(1) $$ADD=\frac{C\times IR}{BW}\times 1000$$

where *ADD* is exposure to fluoride, expressed as μg kg^−1^ day^−1^, *C* is the concentration of fluoride in water (mg L^−1^), *IR* is water ingestion rate, which is set as 0.75, 1, and 2 L d^−1^ for infants, children, and adults, respectively, *BW* refers to body weight, which is set as 5, 10, and 60 kg for infants, children, and adults, respectively. A daily intake of 122 μg kg^−1^ d^−1^ of fluoride might cause fluorosis, while more than 200 μg kg^−1^ d^−1^ of fluoride might lead to skeletal fluorosis issues [15].

Hazard quotient is assessed by Equation (2) as follows:

(2) $$HQ=\frac{ADD}{RfD}$$

where *HQ* is hazard quotient of fluoride, *RfD* is the oral reference dose of fluorine intake in drinking water, which is adopted to be 60 μg kg^−1^ d^−1^ [21]. When the value of *HQ* is larger than 1.00, the non-carcinogenic risk will exceed the acceptable level and a hazard is considered to occur. 

### 2.4. Statistical Analysis

To gain insight into the chemical composition of drinking water sources, obtain more information about dominant indicators influencing the occurrence of fluoride, and analyze the relationships between fluoride and other physicochemical parameters, the multivariate statistical analysis was performed with the statistical package for social sciences (SPSS) version 19 (IBM Corporation, Chicago, USA).

Multivariate statistical analyses including correlation analysis, cluster analysis (CA), principal component analysis (PCA), and factor analysis (FA) are effective tools for analyzing water quality and obtaining meaningful results. The CA method is able to group objects into clusters with high internal (within cluster) homogeneity and high external (between clusters) heterogeneity, usually shown as a dendrogram (tree diagram), which is a visual display of the analysis results with Dlink/Dmax × 100 as the horizontal ordinate. Ward’s method with squared Euclidean distance is widely used in CA to determine the variability. The PCA method extracts principal components by combining original variables linearly to simplify data sets. FA further simplifies the data structure by rotating the axis defined by PCA [19]. In this paper, R-mode cluster analysis (R-CA) was applied to distinguish variables according to the distance between them, and R-mode factor analysis (R-FA) was applied to analyze and organize the large data sets. The comprehensive scores for drinking water sources were obtained by Equation (3) as follows:

(3) $$S=\sum_{i=1}^{n}\sqrt{\lambda_{i}}\times (M_{r}\times M_{ND})$$

where $M_{r}$ is the rotated factor loadings matrix on all the parameters, which is obtained by the rotated factor analysis method, $M_{ND}$ is the normalized data matrix, which is obtained from the measured data in drinking water sources, $\lambda_{i}$ is the eigenvalue of the rotated component matrix, and *n* is the primary component number obtained by R-FA.

## 3. Results and Discussion

### 3.1. Temporal and Spatial Variations of Fluoride Concentrations

**Figure 2:**
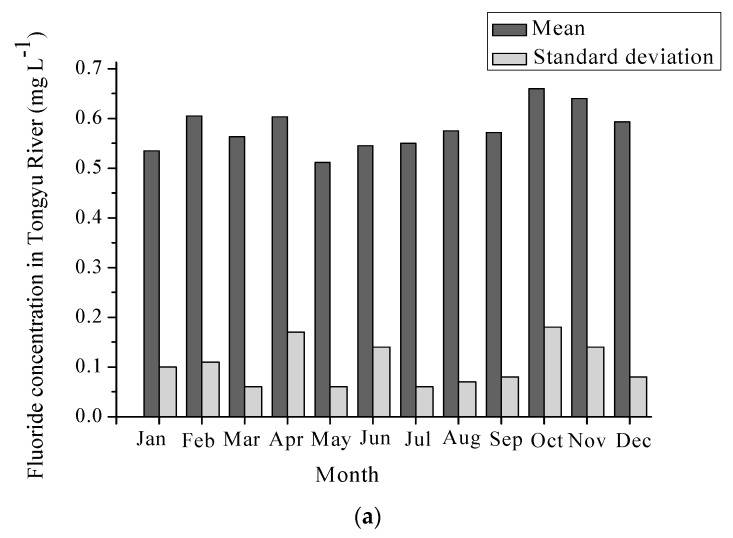

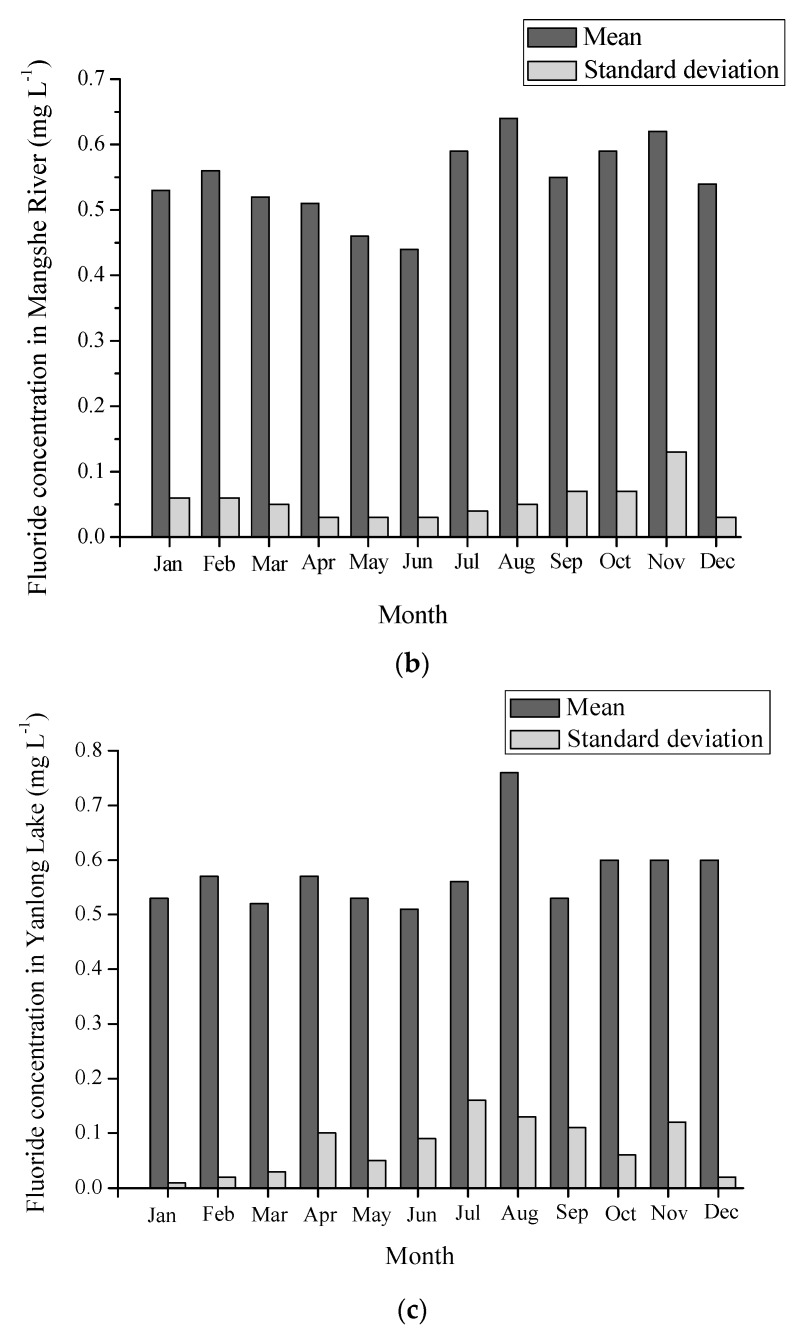
Temporal variations of fluoride concentrations in three drinking water sources (**a**) Monthly fluoride concentration in the Tongyu River. (**b**) Monthly fluoride concentration in the Mangshe River. (**c**) Monthly fluoride concentration in the Yanlong Lake.

The temporal variations of fluoride concentrations in three drinking water sources are shown in Figure 2. The mean fluoride concentrations were 0.58, 0.55, and 0.57 mg L^−1^ in the Tongyu River, the Mangshe River, and the Yanlong Lake, respectively. In the Tongyu River, the variation amplitude was slight, with monthly mean fluoride concentrations ranging from 0.51 to 0.66 mg L^−1^. However, the minimum fluoride concentrations were less than 0.5 mg L^−1^ in almost all of the months except 0.53 mg L^−1^ which occurred in October, 2015 and 0.51 mg L^−1^ which occurred in November, 2013. Furthermore, the fluoride concentration dropped to as low as 0.38 mg L^−1^ in January, 2010. The low concentration of fluoride may induce dental caries. Similarly, the maximum and mean fluoride concentrations also reached higher values in October and November, 2010, which means that fluoride contents may be associated with groundwater with high fluoride concentration since the river is supplied by groundwater in dry seasons (October and November). The fluoride of shallow groundwater in Yancheng City was reported to exceed the standard level of 1.0 mg L^−1^, which led to dental and bone fluorosis [22]. Additionally, a rising number of fluorine-related chemical plants were constructed in the Yancheng districts, which may increase the fluoride content and corresponding health risks through irregular discharge of wastewater into surrounding drinking water sources. As for the Mangshe River, the variety range of maximal, minimal, and mean fluoride content remained almost invariable, which suggests that fluoride in the Mangshe River may come from steady groundwater. However, it is worth noting that the maximal fluorides were lowest (<0.5 mg L^−1^) in May, 2012 and June, 2015, and minimal fluorides were also lowest (<0.5 mg L^−1^) in May, 2015 and June, 2013. The mean fluoride content reached its maximum value of 0.64 mg L^−1^ in August. This supported the claim that large quantities of upstream agricultural nonpoint sources and domestic pollution sources were drained into the Mangshe River, especially in flood seasons. The differences between the Tongyu River and the Mangshe River could be due to the fluoride in the Mangshe River being affected by anthropogenic activity more seriously than the Tongyu River. As for the Yanlong Lake, the maximum fluoride content in July, 2012 and August, 2014 reached 0.80 and 0.95 mg L^−1^, respectively. Since July and August are in flood seasons, large quantities of wastewater probably gave rise to fluoride in the Yanlong Lake.

**Figure 3:**
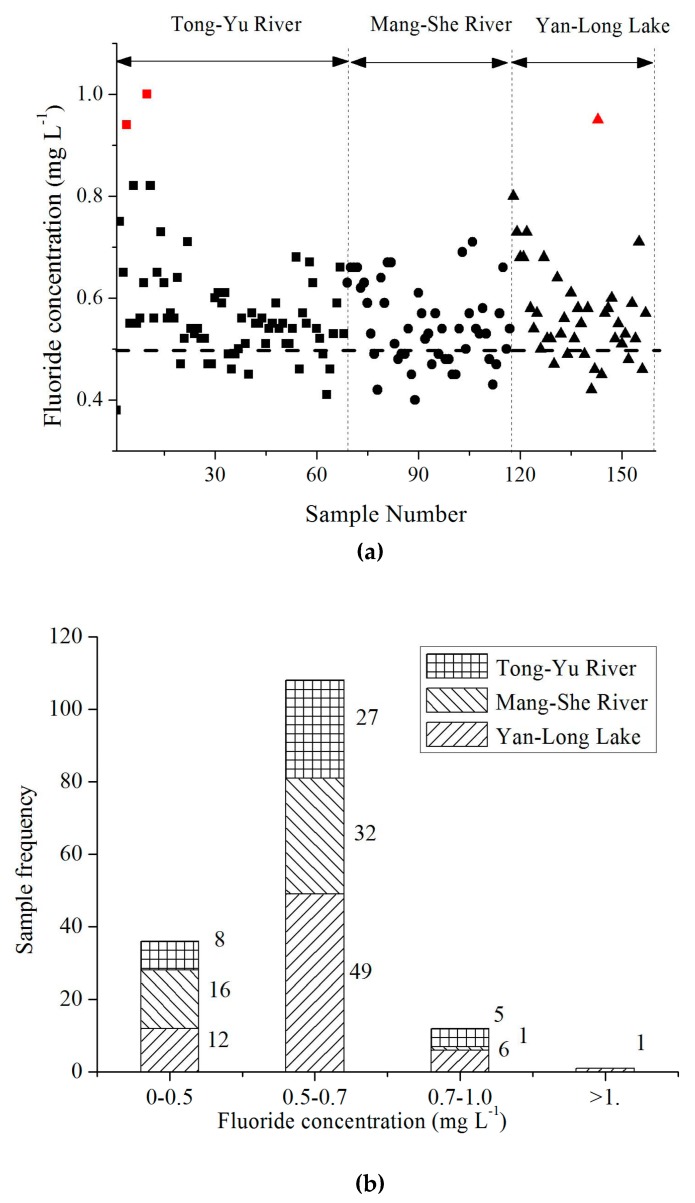
Fluoride contents and frequency distribution in drinking water sources of Yancheng City (**a**) Fluoride contents in drinking water sources of Yancheng City. Note: Dashed line refers to fluoride concentration of 0.5 mg L^−1^, which is the limit to prevent tooth cavity recommended by the WHO. (**b**) Frequency distributions of fluoride concentration in the studied area.

The distributions of fluoride concentration in drinking water sources are presented in Figure 3. In 157 representative water samples, including 68 samples in the Tongyu River, 49 samples in the Mangshe River, and 40 samples in the Yanlong Lake (Figure 3a), the fluoride contents were less than the Chinese sanitary standard for drinking water (1.0 mg L^−1^), the WHO drinking water quality guideline (1.50 mg L^−1^) and U.S. standard (2.0 mg L^−1^). However, in moderate samples (22.9%, n = 157) including 12 samples in the Tongyu River, 16 in the Mangshe River, and 8 in the Yanlong Lake, the fluoride concentrations were less than 0.5 mg L^−1^ recommended by the WHO to prevent tooth cavity (seen in Figure 3b). This may pose an elevated risk of dental decay, especially for children. Moreover, the data for fluoride concentration refer to drinking water sources, not treated finished water from drinking water treatment plants, where fluoride may be removed by coagulation–sedimentation in the traditional purification process. The removal efficiency depends on the type of coagulants, dosage, and sedimentation time. The removal of fluoride increased the risk of dental decay for children. For preventing dental decay, adding fluoride to drinking water is an effective measure. In the U.S., the prevalence rate of dental caries has dropped by 50%–60% in the last 30 years as a result of fluoridation in drinking water. Similar operations could be found in other countries such as England, Russia, Brazil, and Cuba. Therefore, local governments should take measures to prevent dental decay, such as promoting the use of toothpaste with fluoride.

### 3.2. Health Risk Assessment of Fluoride

**Figure 4:**
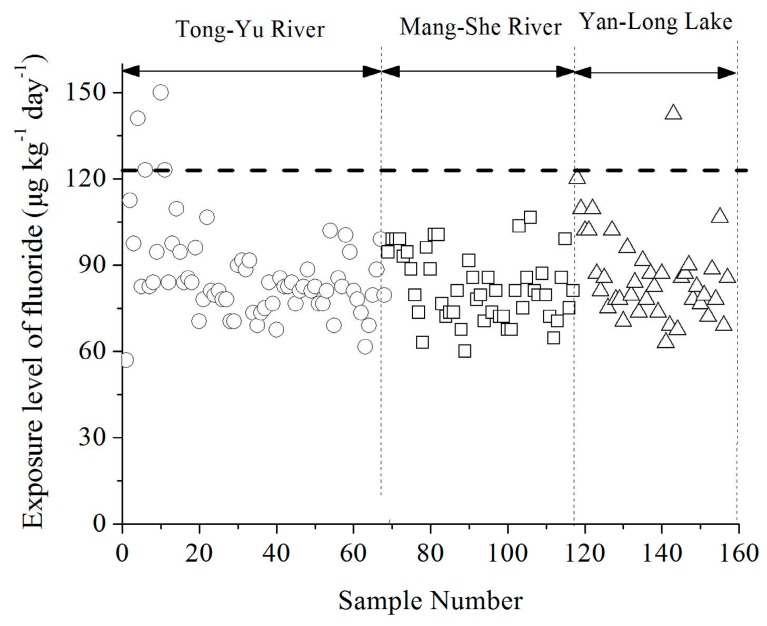
Exposure of fluoride for infants in the three drinking water sources of Yancheng City. Note: Dashed line in Figure 4 refers to the toxicity reference value of 122 μg kg^−1^ d^−1^, which is recommended by Health Canada.

The exposure levels of fluoride (*ADD*) were calculated using Equation (1), and shown in Table S1 (Supporting information). The fluoride exposure level for infants in all the samples were analyzed and shown in Figure 4. The infants of 4 of 68 (5.9%), 0 of 49 (0%), and 1 of 40 (2.5%) for the Tongyu River, Mangshe River, and Yanlong Lake, respectively, had fluoride exposure levels higher than the toxicity reference value (TRVs) as recommended by Health Canada (122 μg kg^−1^ d^−1^), which can cause dental fluorosis issues.

**Figure 5:**
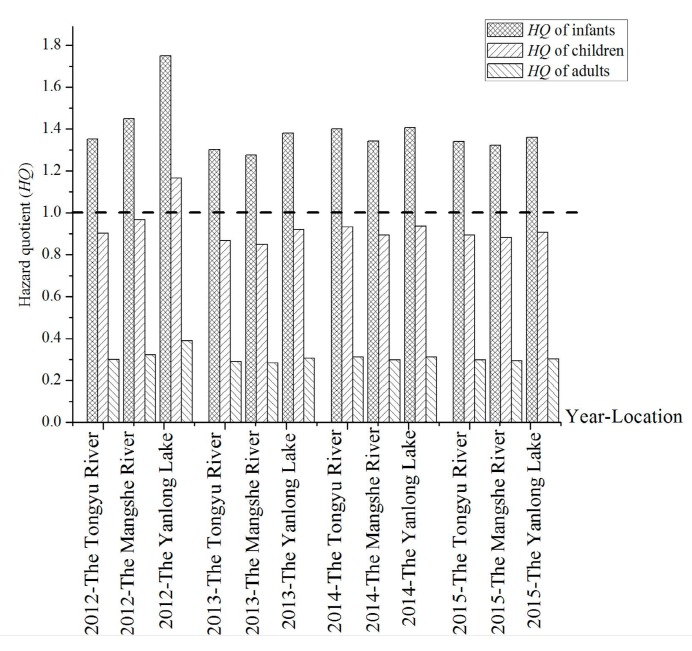
Hazard quotient of fluoride in the three drinking water sources from 2012 to 2015. Note: Dashed line in Figure 5 refers to the hazard quotient (*HQ*) of 1.0, which is the limit recommended by the WHO.

The hazard quotients were calculated using Equation (2) and shown in Figure 5. All the hazard quotients of infants were higher than the recommended value of 1.0 by United States Environmental Protection Agency (USEPA). The results suggested that the infants in Yancheng City suffer from the health risks associated with fluoride. Compared with the Tongyu River and Mangshe River, the hazard quotients were the biggest in Yanlong Lake in 2012 and 2013. In 2012, the hazard quotients showed a significant difference among the three drinking water sources. However, in 2015, the hazard quotients of the three drinking water sources were similar. In addition, the hazard quotients of children and adults were almost always lower than 1.0 except in Yanlong Lake in 2012, which means that the children and adults would not suffer from the harmful effects associated with daily fluoride intake. 

### 3.3. Statistical Analysis

#### 3.3.1. Concentrations of Other Physic-Chemical Parameters

The concentration ranges of the other physicochemical parameters are shown in Table 2. The pH values ranged from 7.2 to 8.4 with an average of 7.6 in the Tongyu River, from 7.3 to 8.7 with an average of 7.7 in the Mangshe River, and from 7.4 to 8.5 with an average of 8.1 in Yanlong Lake. Compared with the WHO recommended limits of pH 6.5–8.5, the drinking water sources of Yancheng City are alkaline. The TDS levels of the three drinking water sources ranged from 122 to 594 mg L^−1^, less than the maximum contamination limit (MCL) of 2000 mg L^−1^ recommended by the WHO (shown in Table 1). However, some of them exceeded the TDS levels of 500 mg L^−1^, which may be unacceptable for consumers because of its taste. High intake of sodium (Na^+^) is harmful for patients suffering from renal, cardiac, and circulatory disease. In Table 2, the concentrations of Na^+^ are less than 200 mg L^−1^, and amounts of K^+^ are less than 250 mg L^−1^, which is listed as potential complaint for taste. The concentrations of Cl^−^ ranged from 35 to 194 mg L^−1^, less than the limit value of 250 mg L^−1^ to prevent heart and kidney diseases from a health perspective. The SO_4_^2−^ concentration in drinking water sources ranged from 23 to 81 mg L^−1^, and high concentration of SO_4_^2−^ may contribute to the taste and the corrosion of water supply distribution systems [5]. The relationship between pH and dissolution of fluoride can be obtained from the Mangshe River and Yanlong Lake. In the Mangshe River, the highest pH value of 8.72 occurred in February, 2012, while the concentration of fluoride was also at high level of 0.63 mg L^−1^. The lowest pH value of 7.28 occurred in September, 2015, while the concentration of fluoride was also at lower level of 0.50 mg L^−1^. Similarly, in Yanlong Lake, the highest pH value of 8.54 occurred in August, 2012, while the concentration of fluoride reached 0.73 mg L^−1^. The lowest pH value of 7.42 occurred in November, 2013, while the concentration of fluoride was also at lower level of 0.49 mg L^−1^. However, in the Tongyu River, the highest pH value of 8.40 occurred in February, 2012, while the concentration of fluoride was only 0.53 mg L^−1^. The lowest pH value of 7.20 occurred in February, 2013, while the concentration of fluoride was also at lower level of 0.49 mg L^−1^. Generally, the results comply with the conclusion that F^−^ contents are associated with the pH values, which is obtained from other literature [23]. The concentrations of Na^+^ in the Tongyu River are higher than that in the Mangshe River and Yanlong Lake, which is the same result obtained from fluoride. The results indicate that higher Na^+^ concentration increases the dissolution of fluoride, which is similar to the findings from other literature [18].

#### 3.3.2. Correlation Analysis

**Figure 6:**
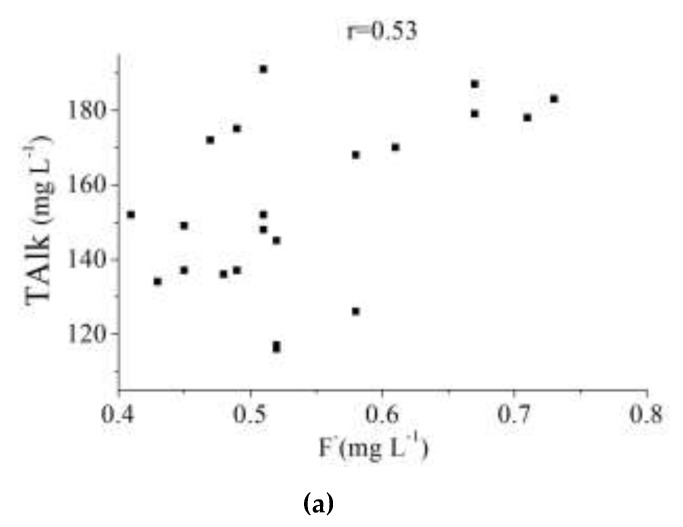

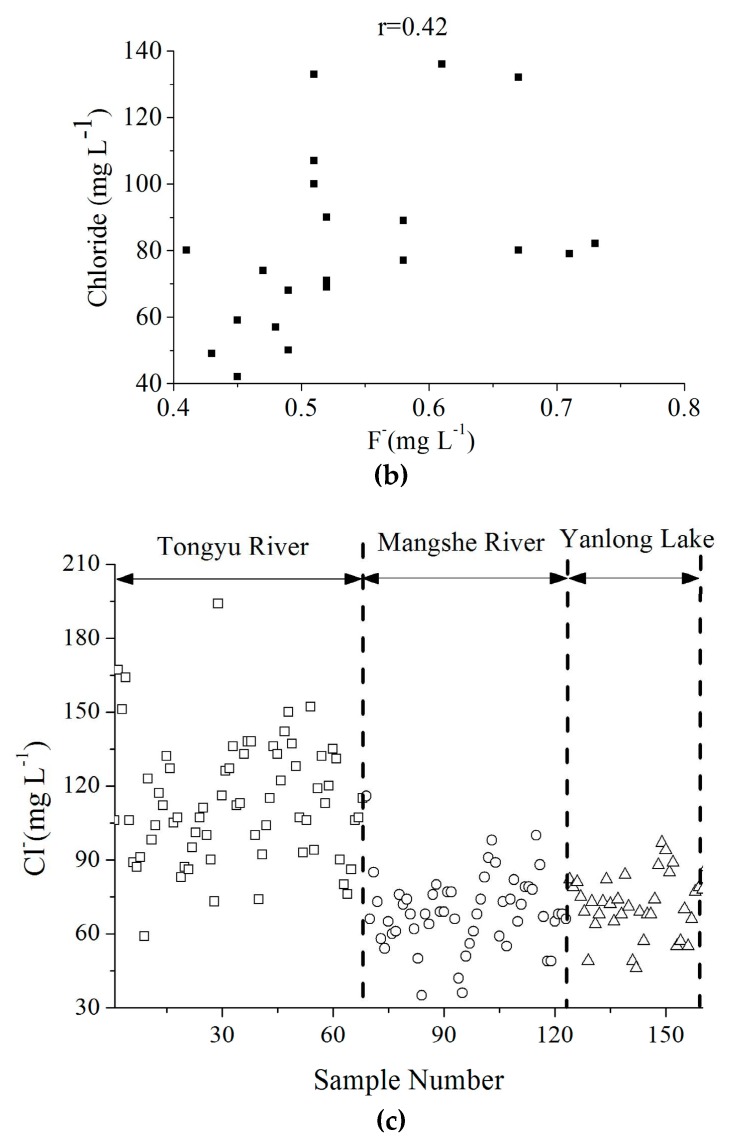
Relationships between F^−^ and TAlk, F^−^ and chloride, and chlorine contents in drinking water sources of Yancheng City (**a**) Correlation between F^−^ and TAlk (**b**) Correlation between F^−^ and chloride (**c**) Chlorine contents in drinking water sources of Yancheng City.

Pearson correlation coefficients for the possible relationships between fluoride and the other monitored physicochemical parameters are summarized in Table 3. There is a positive relationship between fluoride and total alkalinity (TAlk) (r = 0.53), suggesting that fluoride content is associated with the increase of alkalinity in surface water, which is similar to results from other literature [24]. The concentrations of TAlk in the Tongyu River are higher than the Mangshe River and Yanlong Lake, where the concentration of fluoride is also the highest. The correlation between fluoride and TAlk is shown in Figure 6a. Besides TAlk, the statistical relationship between fluoride concentration and Cl^−^ is shown in Figure 6b with positive correlation coefficients of 0.42. The chlorine contents in three drinking water sources are shown in Figure 6c. Similar to fluoride, the chlorine contents in the Tongyu River are obviously higher than those in the Mangshe River and Yanlong Lake. As for the other parameters, the relationship between fluoride and pH, SO_4_^2−^, and TDS are positive with correlation coefficients of 0.39, 0.22, and 0.11, respectively. Moreover, the relationship between fluoride and Na^+^ and K^+^ are negative with correlation coefficients of −0.15 and −0.47, respectively. 

Besides the relationship between fluoride and alkalinity, the correlation analyses of other parameters are described as follows: (1) Chloride is related to sulfate with a correlation coefficient of 0.52; (2) sodium (Na^+^) is observed to have a close relationship with potassium (K^+^) (r = 0.68). The correlation was further interpreted through R-mode cluster analysis and factor analysis.

#### 3.3.3. R-mode Cluster Analysis and Factor Analysis

**Figure 7:**
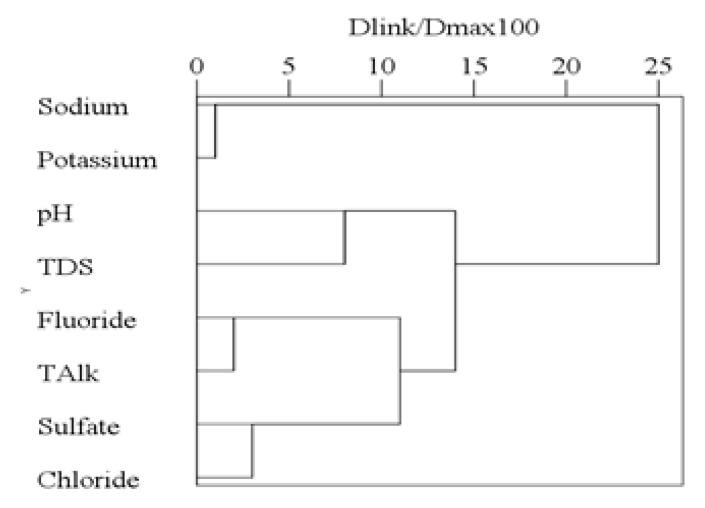
R-Cluster analysis results.

**Figure 8:**
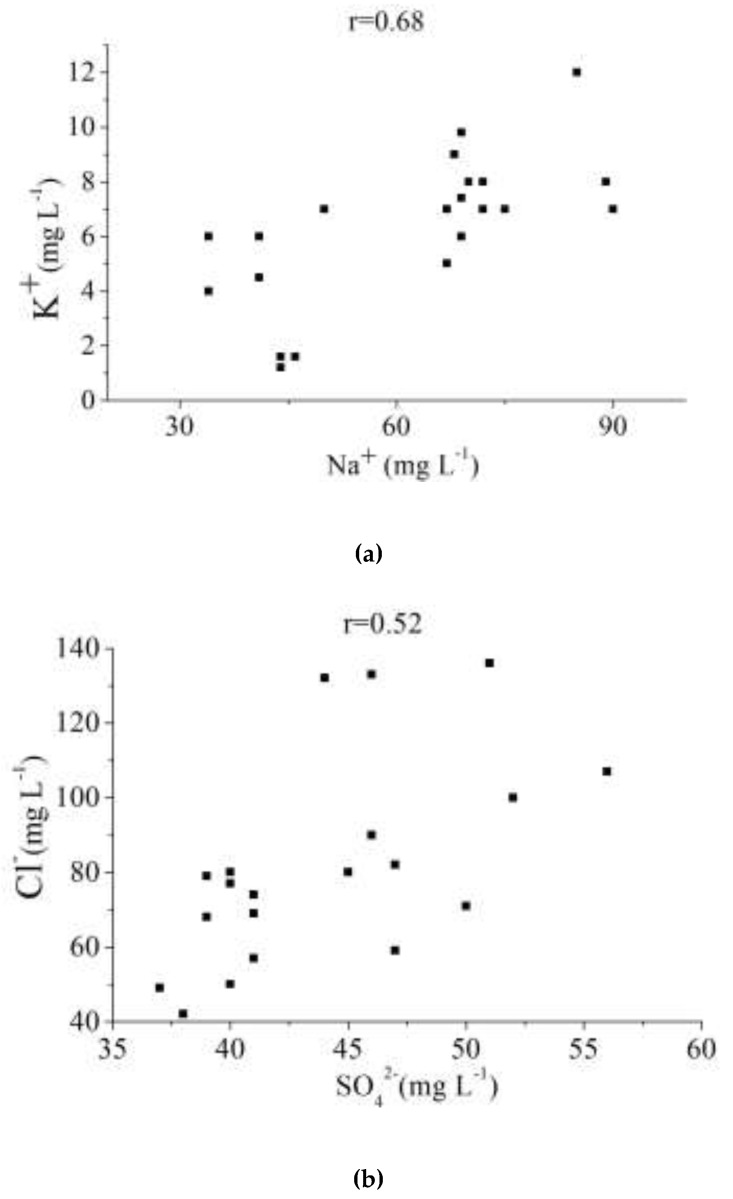
Relationships between Na^+^ and K^+^, and SO_4_^2−^ and Cl^−^ (**a**) Correlation between Na^+^ and K^+^. (**b**) Correlation between SO_4_^2−^ and Cl^−^.

The R-mode cluster analysis was carried out for different physicochemical characters, and the results are shown in a Figure 7. All the variables are grouped into four clusters in a convincing way of Dlink/Dmax × 100 < 10. Fluoride (F^−^) and total alkalinity (TAlk) were grouped into a cluster. The correlation between F^−^ and TAlk is also shown in Table 3 and Figure 6a with a correlation coefficient of 0.53. In addition, the correlation between sodium (Na^+^) and potassium (K^+^) is shown in Table 3 and Figure 8a with a correlation coefficient of 0.68. Similarly, the correlation between sulfate (SO_4_^2−^) and chloride (Cl^−^) is shown in Table 3 and Figure 8b with a correlation coefficient of 0.52. 

**Figure 9:**
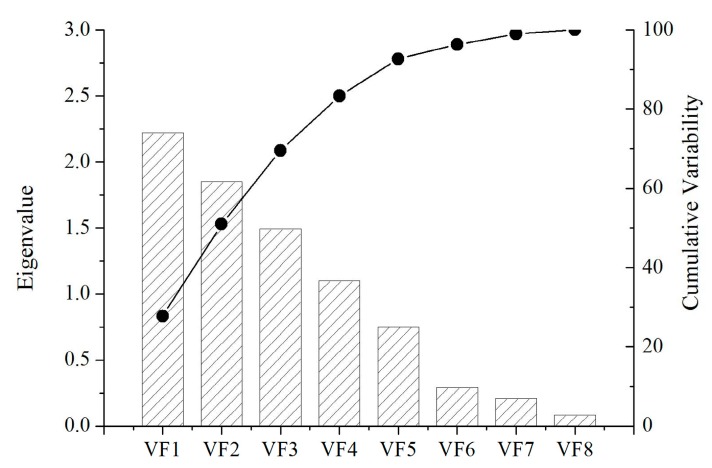
Scree plot of varimax rotated factors.

The qualitative information about clustering behaviors was extracted by R-mode factor analysis. The eigenvalues and cumulative variability of all the eight factors are plotted in Figure 9. The first four factors, having a total variance of 83.31% from all the parameters, correspond to four clusters obtained from the cluster analysis. 

In addition, the rotated factor loadings matrix on all the parameters is expressed in Table 4, including eigenvalues, percentage of variance, and cumulative percentage.

In Table 4, Factor 1 accounted for 27.77% of the total variance and had high loadings of pH (−0.80), Na^+^ (0.81) and K^+^ (0.78), which indicate that the presence of Na^+^ and K^+^ decreased the values of pH. Factor 2, accounting for 23.17% of the total variance, was mainly associated with significantly higher loading of TAlk (0.91) and moderate loading of F^−^ (0.75), which illustrated that F^−^ solubility is TAlk dependent. Factor 3 was an index of the main anions, including SO_4_^2−^ (0.94) and Cl^−^ (0.70), and counted for 18.6% of the total variability, which indicated that the SO_4_^2−^ values of those samples are higher when there is an elevated amount of Cl^−^. Factor 4, counting for 13.77% of the total variability, had a strong relationship with TDS (0.94), which reflected the amount of cations and anions. 

**Figure 10:**
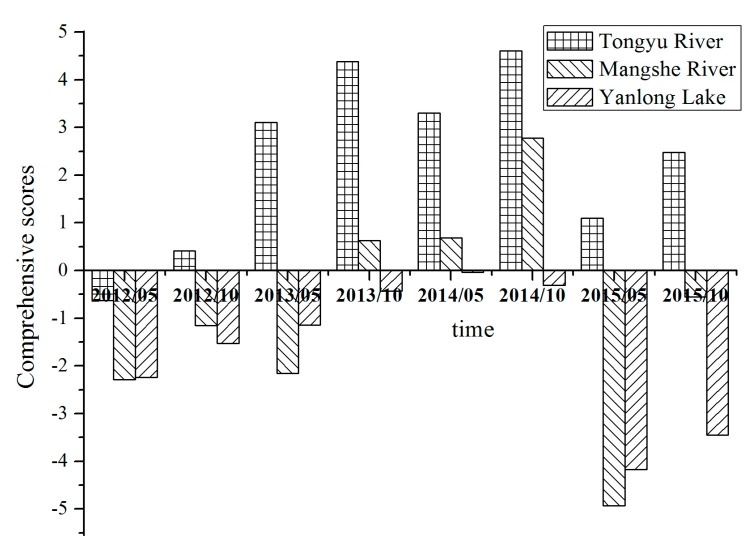
Comprehensive score of drinking water sources.

The comprehensive scores of water sources were calculated using Equation (3), and shown in Figure 10. It is evident that the comprehensive scores of the Tongyu River are significantly greater than those of the Mangshe River and Yanlong Lake, which is due to the fact that the hydrogeological conditions in the Tongyu River were quite different from that in the Mangshe River and Yanlong Lake. 

## 4. Conclusions

This study provided a systematic analysis of fluoride contents in the three drinking water sources of Yancheng City in the Jiangsu province of China. The fluoride concentrations in the drinking water sources were between 0.38 and 1.0 mg L^−1^, which were lower than the recommended value in either the local or international drinking water sources quality guidelines, which means that local people would not suffer from dental fluorosis. The mean fluoride concentrations decreased in the order of Tongyu River > Yanlong Lake > Mangshe River. The results also indicated that the health risks associated with fluoride in infants are higher than children and adults, which might cause dental fluorosis issues, especially for the Tongyu River and Yanlong Lake. However, the fluoride contents less than 0.50 mg L^−1^ unexpectedly occurred in 22.93% of the samples, including 12 samples in the Tongyu River, 16 in the Mangshe River and 8 in Yanlong Lake, which increase the risk of tooth cavities. Therefore, continuous and closer monitoring work should be performed by the local water management department. In addition, to prevent dental caries, corresponding measures should be taken to increase fluoride content through tea, fluoridated toothpaste, and fluoridated salt, etc., especially for the Mangshe River.

The correlation analysis among other physicochemical parameters indicated that relationships existed between F^−^ and TAlk (r = 0.53), SO_4_^2−^ and Cl^−^ (r = 0.52), and Na^+^ and K^+^ (r = 0.68), which was also verified by R-mode cluster analysis and factor analysis. Four factors were extracted by R-mode factor analysis, which represent cation, fluoride content, anion, and TDS, respectively. The concentrations of TAlk in the Tongyu River were higher than Yanlong Lake and the Mangshe River, which is in accordance with the fluoride level in three drinking water sources. The results of comprehensive assessment illustrated that physicochemical character of the Tongyu River was significantly different from that of the Mangshe River and Yanlong Lake, which is primarily due to different hydrogeological conditions.

## Figures and Tables

**Table 1:** Limit specified of physicochemical parameters in drinking water sources (CJ3020-93).

Parameters	Limit	Analytical Methods	Detection Criteria
Potassium (K^+^)	N/A	Flame photometer	(MHPRC, GB/T 5750.6-2006)
Sodium (Na^+^)	N/A	Flame photometer	(MHPRC, GB/T 5750.6-2006)
Fluoride (F^−^)	1.0	Ion selective electrode	(MHPRC, GB/T 5750.5-2006)
pH	6.5–8.5	pH-meter	(MHPRC, GB/T 5750.4-2006)
Chloride (Cl^−^)	250	Titrimetric	(MHPRC, GB/T 5750.5-2006)
Total Alkalinity (TAlk)	N/A	Titrimetric	(EPBPRC, 2002)
Sulfate (SO_4_^2−^)	250	Spectrophotometric	(MHPRC, GB/T 5750.5-2006)
Total Dissolved Solids (TDS)	1000	Gravimetric	(MHPRC, GB/T 5750.4-2006)

Note 1: Units for all parameters are mg L^−1^ except pH. Note 2: N/A: not available.

**Table 2:** Other physicochemical parameters in three drinking water sources of Yancheng City.

Drinking Water Sources		pH	SO_4_^2−^	Cl^−^	TDS	TAlk	Na^+^	K^+^
Tongyu River	Max.	8.40	81	194	594	218	85	12
	Min.	7.20	23	59	269	117	44	1.2
	Mean	7.57	49	114	411	167	65	6.9
Mangshe River	Max.	8.72	64	116	523	187	90	7.4
	Min.	7.28	25	35	122	123	34	1.6
	Mean	7.65	43	69	380	158	60	5.7
Yanlong Lake	Max.	8.54	61	97	512	183	89	9
	Min.	7.42	24	46	231	116	34	1.6
	Mean	8.12	44	71	369	156	60	6.4

Note: Units for all parameters are mg L^−1^ except pH.

**Table 3:** Correlation matrix of parameters in the studied area.

Parameters	pH	F^−^	SO_4_^2−^	Cl^−^	TDS	TAlk	Na^+^	K^+^
pH	1.00							
F^−^	0.39	1.00						
SO_4_^2−^	0.19	0.22	1.00					
Cl^−^	−0.23	0.42	0.52 *	1.00				
TDS	0.09	0.11	0.22	−0.06	1.00			
TAlk	−0.17	0.53 *	−0.12	0.44	−0.22	1.00		
Na^+^	−0.46	−0.15	−0.05	0.24	−0.31	0.32	1.00	
K^+^	−0.32	−0.47	−0.18	0.00	−0.07	−0.10	0.68 *	1.00

Note: “*” refers to relative coefficients higher than 0.5.

**Table 4:** Varimax rotated R-mode factor loadings on all the parameters.

Parameters	VF1	VF2	VF3	VF4
pH	−0.80 *	−0.14	0.15	−0.19
F^−^	−0.45	0.75 *	0.25	0.04
SO_4_^2−^	−0.15	−0.06	0.94 *	0.13
Cl^−^	0.24	0.55	0.70 *	−0.01
TDS	−0.09	−0.09	0.12	0.94 *
TAlk	0.16	0.91 *	−0.07	−0.16
Na^+^	0.81 *	0.14	0.12	−0.35
K^+^	0.78 *	−0.32	0.01	−0.15
Eigenvalue	2.22	1.85	1.49	1.10
% of the total variance	27.77	23.17	18.60	13.77
% of cumulative	27.77	50.94	69.54	83.31

Note: “*” refers to absolute values higher than 0.7.

## Supplementary Materials

The following are available online at https://www.mdpi.com/1660-4601/16/6/1030/s1.
