# Supplementary material for: Evaluation of Physicochemical Characteristics in Drinking Water Sources Emphasized on Fluoride: A Case Study of Yancheng, China
Source: Int J Environ Res Public Health. 2019 Mar 21;16(6):1030. doi: 10.3390/ijerph16061030 (PMC6466306; doi:10.3390/ijerph16061030)
Supplement: Supplementary file 1 [file ijerph-16-01030-s001.pdf]

Table S1 Fluoride exposure levels in three water sources of Yancheng City. (ug kg^{-1} day^{-1}).

		Tongyu River				Mangshe River				Yanlong Lake			
		F^-	ADD_1	ADD_2	ADD_3	F^-	ADD_1	ADD_2	ADD_3	F^-	ADD_1	ADD_2	ADD_3
2012	Max	0.61	92	61	20	0.67	101	67	22	0.80	120	80	27
	Min	0.47	71	47	16	0.42	63	42	14	0.58	87	58	19
	Mean	0.54	81	54	18	0.58	87	58	19	0.70	105	70	23
2013	Max	0.57	86	57	19	0.61	92	61	20	0.68	102	68	23
	Min	0.45	68	45	15	0.4	60	40	13	0.47	71	47	16
	Mean	0.52	78	52	17	0.51	77	51	17	0.55	83	55	18
2014	Max	0.68	102	68	23	0.71	107	71	24	0.95	143	95	32
	Min	0.46	69	46	15	0.45	68	45	15	0.42	63	42	14
	Mean	0.56	84	56	19	0.54	81	54	18	0.56	84	56	19
2015	Max	0.66	99	66	22	0.66	99	66	22	0.71	107	71	24
	Min	0.41	62	41	14	0.43	65	43	14	0.46	69	46	15
	Mean	0.54	80	54	18	0.53	79	53	18	0.54	82	54	18

Notes: ADD_1, ADD_2, and ADD_3 refer to fluoride exposure level for infants, children, and adults, respectively.
